# Notes and Notices

**Published:** 1894-07

**Authors:** 


### NOTES AND NOTICES.

THE YALE SURGICAL CHAIR, which has been advertised in this journal for the last three or four years, was exhibited at the World's Fair at Chicago in 1893. It drew the highest award at that great exposition. A sample of it may be seen in this office.

